# The association between comorbidities and self-care of heart failure: a cross-sectional study

**DOI:** 10.1186/s12872-023-03166-2

**Published:** 2023-03-27

**Authors:** Kyoung Suk Lee, Debra K. Moser, Kathleen Dracup

**Affiliations:** 1 College of Nursing, the Research Institute of Nursing Science, Seoul National University, 103 Daehak-ro, Jongno-gu, Seoul, 03080 South Korea; 2 College of Nursing, University of Kentucky, Lexington, USA; 3 School of Nursing, University of California San Francisco, San Francisco, CA USA

**Keywords:** Comorbidity, Heart failure, Self-care, Patient compliance

## Abstract

**Background:**

Because heart failure (HF) is a debilitating chronic cardiac condition and increases with age, most patients with HF experience a broad range of coexisting chronic morbidities. Comorbidities present challenges for patients with HF to successfully perform self-care, but it is unknown what types and number of comorbidities influence HF patients’ self-care. The purpose of this study was to explore whether the number of cardiovascular and non-cardiovascular conditions are associated with HF self-care.

**Methods:**

Secondary data analysis was performed with 590 patients with HF. The number of cardiovascular and non-cardiovascular conditions was calculated using the list of conditions in the Charlson Comorbidity Index. Self-care was measured with the European HF self-care behavior scale. Multivariable linear regression was performed to explore the relationship between the types and number of comorbidities and self-care.

**Results:**

Univariate analysis revealed that a greater number of non-cardiovascular comorbidities was associated with poorer HF self-care(β=-0.103), but not of more cardiovascular comorbidities. In the multivariate analysis, this relationship disappeared after adjusting for covariates. Perceived control and depressive symptoms were associated with HF self-care.

**Conclusion:**

The significant relationship between the number of non-cardiovascular comorbidities and HF self-care was not independent of perceived control and depressive symptoms. This result suggests a possible mediating effect of perceived control and depressive symptoms on the relationship between HF self-care and the number and type of comorbidities.

## Introduction

Because heart failure (HF) is a debilitating chronic cardiac condition and increases with age, most patients with HF experience a broad range of coexisting chronic morbidities including cardiac and non-cardiac diseases [1–3]. In Tisminetzky and colleagues’ study (2018) with 114,553 community dwellers with HF, patients had six chronic conditions, on average, with 17.4% having nine or more of 26 possible chronic conditions [2]. In their study, the most frequently observed chronic conditions were, in descending order, hypertension, dyslipidemia, visual impairment, and chronic kidney disease.

Self-care is conceptualized as a naturalistic decision-making process to maintain physiological stability, facilitate symptom perception, and take action to improve symptoms when symptoms are changed [4]. However, comorbidities present unique challenges for patients with HF to perform self-care, as they may deal with overlapping symptom profiles between HF and other chronic conditions. For example, shortness of breath and fatigue, typical symptoms of HF, are also common symptoms of chronic obstructive pulmonary disease. Thus, patients with comorbid conditions may find it difficult to interpret their symptom experiences and respond appropriately [5, 6]. Because patients are also asked to simultaneously adhere to multiple self-care regimens for their comorbidities and HF, they need to develop strategies for a variety of therapeutic regimens for multiple conditions. For HF patients with other cardiovascular conditions, adhering to recommended self-care regimens can be relatively uncomplicated because some recommended regimens (e.g., low sodium diet) are common across those conditions. Thus, it may be that engaging HF self-care is less difficulty for HF patients with cardiovascular comorbidities compared to those with non-cardiovascular comorbidities.

Although it is evident that comorbidity complicates HF patients’ self-care, little is known about who may be at risk for poor HF self-care when they also have comorbid conditions. Kerr and colleagues first classified comorbidities in patients with diabetes into “concordant” conditions, which represented condition with an identical overall pathophysiologic risk profiles to diabetes, and “discordant” conditions, which were not directly related to diabetes in either their pathogenesis or management and did not share underlying risk factors (predisposing factors)[7]. They found that types of comorbidities were associated with self-care ability of patients with diabetes in addition to the number of comorbid conditions [7]. Their findings stimulated further research related to comorbidities of diabetes. Similar to the diabetes population, considering the types and number of conditions is also important for patients with HF. Therefore, the purpose of this study was to enhance our understanding of the impact of the number of cardiovascular and non-cardiovascular conditions on HF self-care. We hypothesized that patients with more cardiovascular comorbidities would have better HF self-care than those with fewer cardiovascular comorbidities, and that patients with more non-cardiovascular comorbidities would have poorer HF self-care than those with fewer non-cardiovascular comorbidities.

## Methods

### Study design and sample

The present study is a secondary analysis of the baseline data in a randomized, clinical trial designed to study the effects of an educational intervention on the prognosis and quality of life for patients with HF who lived in rural areas in the United States from March, 2007 to January, 2013 (www.ClincalTrials.gov-NCT00415545) [8]. Patients were eligible if they were older than 18 years old, and had a confirmed diagnosis of HF, history of hospitalization due to HF within the last six months before enrollment, and intact cognition. Exclusion criteria included having life-threatening comorbid conditions such as active cancer treatment, were non-English speaking, or were living in a nursing home or assisted living facility. The parent study was approved by the Institutional Review Boards of all three participating institutions and conformed to the ethical principles outlined in the Declaration of Helsinki. Participants provided signed, written informed consent. The approval of the secondary data analysis was approved by the Institutional Review Boards at the affiliated institute (IRB # 84922).

### Procedures

The detailed study procedure of the parent study is described elsewhere [8]. After giving written, signed informed consent, patients completed structured questionnaires to gather sociodemographic data and information on self-care, perceived control, and depressive symptoms. Clinical information (e.g., left ventricular ejection fraction) was collected through a medical record review and patient interview. The Charlson Comorbidity Index [9] was used to assess comorbid conditions, and was completed based on patients’ self-report. For the current study, we examined HF self-care of 590 patients who provided data on all of the variables of interest out of 602 patients enrolled in the parent study. There were no significant demographic or clinical differences in patients who were included and those who were excluded in this study.

### Measurements

#### Outcome variable

##### Self-care

Self-care was measured with the European HF self-care behavior scale (EHFScB-9) [10]. This scale consists of 9 items, which are rated on a five-point scale from 1 (“completely agree”) to 5 (“do not agree at all”). Total scores can range from 9 to 45, with higher scores indicating poorer self-care. However, for ease of interpretation, a new scoring method was suggested and validated by Vellone and colleagues (2014) [11]. The new scoring method includes standardizing the scores ranging from 0 to 100, with higher scores representing better self-care. The standardized score is computed by subtracting nine from the item total and multiplying by 2.7777 after reverse-scoring. We used the standardized scores of the EHFScB-9.

#### Explanatory variable

##### Comorbid conditions

Patients’ comorbid condition profiles were expressed as the number of cardiovascular and non-cardiovascular conditions. Medical record reviews were performed to collect the data on comorbid conditions listed in the Charlson Comorbidity Index [9]. However, of the 19 conditions included in Charlson Comorbidity Index, dementia and AIDS/HIV were excluded because dementia was the exclusion criterion of the parent study, and no one reported having AIDS/HIV. A total of 16 conditions were categorized into cardiovascular (4 conditions) and non-cardiovascular (12 conditions) conditions. Cardiovascular conditions included myocardial infarction, peripheral vascular disease, cerebrovascular disease, and hemiplegia. Non-cardiovascular conditions included the following: renal diseases, diabetes with and without end organ damage, chronic pulmonary disease, peptic ulcer, connective tissue diseases, mild liver disease, moderate to severe liver disease, solid tumor with and without metastasis, leukemia, and lymphoma.

#### Covariates

##### Perceived control

Perceived control was measured using the 8-item Control Attitudes Scale-Revised [12]. Patients were asked to rate their sense of control over their cardiac problems on a 5-point scale (1 = totally disagree, 5 = totally agree). Total scores range from 8 to 40, with a higher score indicating greater levels of perceived control.

##### Depressive symptoms

The Patient Health Questionnaire-9 was used to measure depressive symptoms [13, 14]. The items of this measure correspond to the 9 diagnostic criteria for major depressive disorders in the Diagnostic and Statistical Manual of Mental Disorder IV. Patients were asked to rate each item from 0 (not at all) to 3 (nearly every day) points. Total scores can range from 0 to 27, with higher scores indicating higher levels of depressive symptoms. Scores of 10 or greater indicate a clinically significant levels of depressive symptoms [13].

##### Demographic and clinical variables

A self-reported questionnaire was used to collect data on sociodemographic information. The New York Heart Association functional class was determined by trained research nurses based on careful patient interviews. Medical record reviews were also conducted to collect clinical information. Patients’ left ventricular ejection fraction was defined as reduced (40% or below), mid-range (40–49%), and preserved left ventricular systolic function (50% or above).

### Statistical analyses

Descriptive statistics were used to summarize the characteristics of our sample. After performing the univariate linear regression, multivariate linear regression was conducted to examine the relationship between the number of cardiovascular and non-cardiovascular comorbid conditions and self-care after adjusting for covariates (i.e., age, gender, race, living arrangement, employment status, education level, etiology of HF, reduced left ventricular systolic function, perceived control, and depressive symptoms).

Three additional analyses were also conducted with the three aims. The first aim was to explore the relationship between HF self-care and the types of comorbid conditions (i.e., cardiovascular and non-cardiovascular comorbid conditions) without considering the number of conditions. For this analysis, patients were grouped as either having or not having cardiovascular and non-cardiovascular comorbid conditions. The second aim was to explore the relationship between HF self-care and the number of comorbid conditions regardless of the types. The third aim was to explore the association between HF self-care and the Charlson Comorbidity Index scores, which are the sum of the weights of each condition. Data were analyzed with IBM SPSS version 25 (IBM Corporation, Armonk, NY). The significance level was set at *p*-value < 0.05.

## Results

### Sample characteristics

A total of 590 patients were included in this study. The average age of the patients was 66 years (*SD*: 0.31), and more than half of the patients were 65 years or older (57.0%) (Table 1). The majority of the patients were male, white, non-employed or retired, lived with someone, and had less than a high school education level. About one-third of the patients (35.4%) were in New York Heart Association functional class III/IV, and less than a half of the patients (47.7%) had ischemic etiology of HF. Slightly less than one-third of the patients (30.8%) had the Patient Health Questionnaire-9 scores of 10 or above, indicating clinically significant depressive symptoms. The average scores of EHFScB-9 were 69.7 (*SD*: 19.2).

**Table 1:** Sample characteristics (N = 590)

	Total
Age, years	66.0 (13.0)
Female	241 (40.8%)
White	525 (89.0%)
Living alone	137 (23.2%)
Employed	86 (14.6%)
High school above education	284 (48.1%)
New York Heart Association function class III/IV	209 (35.4%)
Categories of heart failure	
Reduced ejection fraction (< 40% of LVEF)	301 (51.0%)
Mid-rage ejection fraction (40–49% of LVEF)	108 (18.3%)
Preserved ejection fraction (≥ 50% of LVEF)	181 (30.7%)
Ischemic etiology of heart failure (n = 589)	281 (47.7%)
Medications (n = 589)	
ACEI or ARB	439 (74.4%)
Beta blockers	474 (80.3%)
Diuretic	495 (83.9%)
Perceived control	29.4 (5.0)
Depressive symptoms	7.3 (6.4)
Self-care	69.7 (19.2)

Note. LVEF = left ventricular ejection fraction, ACEI = angiotensin-converting enzyme inhibitor, ARB = angiotensin receptor blocker

Values are n (%) or mean (Standard deviation)

### Comorbid conditions

On average, patients had two comorbid conditions (*SD*: 1.45), with a range of 0 to 7 (Table 2). About half of the patients (49.3%) had both cardiovascular and non-cardiovascular comorbid conditions. Of the cardiovascular comorbid conditions, myocardial infarction (50.5%) was most frequently reported, followed by peripheral vascular disease (32.3%), cerebrovascular disease (14.6%), and hemiplegia (1.2%). Of the non-cardiovascular comorbid conditions, frequently reported conditions were chronic pulmonary disease (33.9%), diabetes without end organ damage (33.1%), and peptic ulcer (14.4%).

**Table 2:** Description of the comorbid conditions (N = 590)

	Total
Number of total comorbid conditions, mean (SD)	2.1 (1.45)
Number of cardiovascular conditions, mean (SD)	0.98 (0.87)
Myocardial infarction	298 (50.5%)
Peripheral vascular disease	190 (32.2%)
Cerebrovascular disease	86 (14.6%)
Hemiplegia	7 (1.2%)
Number of non-cardiovascular conditions, mean (SD)	1.2 (0.99)
Chronic pulmonary disease	200 (33.9%)
Diabetes without end organ damage	195 (33.1%)
Peptic ulcer	85 (14.4%)
Diabetes with end organ damage	52 (8.8%)
Connective tissue diseases	41 (6.9%)
Solid tumor without metastasis	29 (4.9%)
Renal dysfunction	27 (4.6%)
Mild liver disease	13 (2.2%)
Lymphoma	7 (1.2%)
Solid tumor with metastasis	7 (1.2%)
Moderate to severe liver disease	1 (0.2%)
Leukemia	1 (0.2%)

Note. SD = standard deviation

Values are n (%), otherwise being indicated

### Relationship between the comorbid conditions and self-care

In the univariate linear regression model, the number of non-cardiovascular comorbid conditions was statistically significantly associated with self-care, but not the number of cardiovascular comorbid conditions (Table 3). Patients with a greater number of non-cardiovascular comorbid conditions were more likely to have poorer self-care (standardized coefficient: -0.103; 95% confidence interval=-3.593 – -0.405; *p*-value = 0.014).

**Table 3:** The association between comorbid conditions and heart failure self-care (N = 590)

	B	β	p-value	95% confidence interval
Univariate analysis				
Number of cardiovascular comorbid conditions	0.098	0.004	0.915	-1.719, 1.915
Number of non-cardiovascular comorbid conditions	-1.999	-0.103	0.014	-3.593, -0.405
Multivariate analysis				
Number of cardiovascular comorbid conditions	0.563	0.026	0.549	-1.281, 2.407
Number of non-cardiovascular comorbid conditions	-0.992	-0.051	0.233	-2.625, 0.641
Age	-0.040	-0.027	0.543	-0.168, 0.088
White	-4.822	-0.079	0.053	-9.7, 0.057
Living alone	-0.022	0.000	0.991	-3.651, 3.607
Employed	-3.323	-0.061	0.159	-7.956, 1.31
High school above education	0.063	0.002	0.968	-3.013, 3.14
New York Heart Association functional class III/IV	-1.578	-0.039	0.350	-4.895, 1.738
Categories of heart failure				
Reduced ejection fraction (< 40% of LVEF), reference group	1			
Mid-rage ejection fraction (40–49% of LVEF)	-2.921	-0.059	0.169	-7.091, 1.249
Preserved ejection fraction (≥ 50% of LVEF)	-2.413	-0.058	0.192	-6.042, 1.215
Perceived control	0.557	0.146	0.001	0.22, 0.895
Depressive symptoms	-0.493	-0.165	< 0.001	-0.767, -0.219

Note. LVEF = left ventricular ejection fraction

Model p-values for univariate and multivariate models 0.045 and < 0.001, respectively

In the multivariate linear regression model, neither the number of cardiovascular nor non-cardiovascular comorbid conditions was statistically significantly associated with self-care after adjusting for covariates. Among the covariates entered in the model, perceived control and depressive symptoms care (standardized coefficient: 0.146 and − 0.165; 95% CI = 0.22–0.895 and − 0.767 – -0.219; *p*-value = 0.001 and < 0.001 respectively) were statistically significantly associated with HF self-care.

### Additional analyses

In the both univariate and multivariate linear regression models, types of comorbid conditions were not statistically significantly associated with self-care. Identical results were found when the number of total comorbid conditions regardless of their type was entered in the both univariate and multivariate linear regression models. When Charlson Comorbid Index scores were entered to explore the relationship between this score and self-care in the univariate and multivariate linear regression model, self-care was not related to the Charlson Comorbid Index scores.

## Discussion

We explored the relationship between HF self-care and comorbid conditions with the underlying assumption that both the types and the number of comorbid conditions could influence patients’ adherence to HF self-care activities. Our findings showed that a greater number of non-cardiovascular comorbidities was associated with patients’ adherence to HF self-care activities, but not the number of cardiovascular comorbidities. Our assumption was further supported by the results of our additional analyses. Neither the number of total comorbid conditions or the types of comorbid conditions were associated with HF self-care. However, the significant relationship between self-care and the types and number of comorbidities disappeared when the covariates were entered in the model. Our results imply that perceived control and depressive symptoms may be mediators of the relationship between comorbidities and HF self-care.

The presence of multiple comorbid conditions can substantially increase patients’ treatment burden because patients are required to manage a variety of self-care activities for both HF and comorbidities (e.g., medication management, clinic appointments, and lifestyle modifications), reconcile information from multiple clinicians, and monitor and distinguish between HF symptoms and those of other conditions. Several studies have indicated that patients’ day-to-day decisions related to HF self-care are complicated when multiple conditions co-exist with HF, as some comorbid conditions may present competing demands for performing HF self-care activities [6, 15, 16]. Our findings expand previous findings because our study showed the importance of considering the types and number of comorbid conditions in HF self-care, which goes beyond a simple count or burden of comorbidities.

The univariate analysis revealed a significant relationship between the number of non-cardiovascular comorbidities and HF self-care, and the non-significant association between the number of cardiovascular comorbidities and HF self-care. To the best of our knowledge, our study is the first to explore the relationship between self-care and the types and number of comorbid conditions in HF, which makes it difficult to compare our study findings with previous findings. The relationship between individual comorbidities (e.g., peripheral artery disease, diabetes, and renal disease) and self-care has been explored in a limited number of the studies. However, consistent relationships between individual comorbidities and self-care were not found across the studies [17, 18].

Because of the lack of relevant previous studies, it was difficult to interpret our results. However, we may be able to explain the significant association between self-care and the number of non-cardiovascular comorbidities based on three reasons. One reason for this finding could be related to patients’ limited capacity to simultaneously deal with HF and non-cardiovascular conditions. To successfully engage in self-care, patients need a comprehensive understanding of comorbidities and HF. However, because recommended therapeutic regimens for non-cardiovascular conditions can vary widely from that of HF, patients with HF and non-cardiovascular conditions may be confused and have challenges understanding the variety of self-care activities in a coherent manner. For example, in a previous qualitative study, one patient with HF and diabetes did not weigh himself because the patient believed that monitoring weight daily was harmful to losing weight for his diabetes [6].

The second reason may be that patients give lower priority to HF self-care than to self-care for non-cardiovascular comorbidities, although we did not directly ask patients what priority they gave to HF self-care versus their other comorbidities. Patients with diabetes as well as more cardiac comorbidities and discordant comorbidities (conditions that are not directly related to diabetes) were likely to give lower priority to diabetes self-care [7], which is in line with our finding. In a previous review, various internal and external factors were reported to affect the prioritization process [19]. One of the internal factors was how well the disease was controlled, and if well-controlled conditions were given lower priority. Because the majority of our sample was in New York Heart Association functional class I/II, indicating no or mild symptoms that minimally interfered with their daily activities, most patients in our study may have believed that HF was under control so other comorbidities were given higher priority than HF. However, we did not have evidence that non-cardiovascular comorbidities were given higher priority than cardiovascular comorbidities as we did not collect this information.

The last reason may be the fragmented care for patients with multiple chronic conditions. Some comorbidities are addressed in the HF management guidelines (e.g., stroke, diabetes, and kidney diseases) [3, 20, 21]. However, the depth of the recommendations and comorbid conditions included in the guidelines are not consistent, and the AHA/ACC/HFSA guideline for the management of HF noted the difficulty of suggesting specific recommendations for some comorbid conditions, including most non-cardiovascular conditions, due to the lack of current evidence [3]. Therefore, it would be difficult for HF specialists including physicians and nurses to explain HF self-care in relation to patients’ non-cardiovascular comorbidities. Similarly, specialists of other discordant conditions may not address HF and may give conflicting information about HF management and the other conditions.

Although we found a significant association between the number of non-cardiovascular conditions and HF self-care in the univariate regression model, this relationship did not remain when covariates were included in the multivariate regression model. Of the covariates, perceived control and depressive symptoms were significantly related to HF self-care. This result has been consistently reported in numerous HF studies [22–24]. Studies have reported that patients with a greater number of chronic conditions were at risk for lower levels of self-efficacy (which is a related concept to perceived control), and higher levels of depressive symptoms in patients recruited in primary care settings or the community [25–27]. From these findings, we suspect that the relationship between the types and number of comorbidities and HF self-care is mediated by patients’ perceived control and depressive symptoms. However, some investigators have suggested a moderating role of comorbidities on the relationship between HF self-efficacy and HF self-care [6, 28], but the findings in previous studies have been inconsistent. Thus, further research is needed to clarify the role of perceived control (or self-efficacy) and depressive symptoms on the relationship between self-care and comorbidities.

## Limitations

Although this study highlights the importance of considering the types and number of comorbidities when investigating HF self-care, our study has several inherent limitations to be noted by using the existing data. The data collection for comorbidity using the Charlson Comorbidity Index was performed to suite the primary purpose of the original investigators. The list of chronic conditions included in the Charlson Comorbidity Index may not be comprehensive. Although the Charlson Comorbidity Index is one of the most popular instruments to measure comorbidities[29] and includes some of the prevalent comorbidities in the HF population [1], the instrument was originally developed to estimate one-year mortality of hospitalized patients and was validated with women receiving treatment for breast cancer [9]. Thus, some authors have raised concerns about using the Charlson Comorbidity Index for this area of research [6]. Our study sample was limited to patients with HF living in rural areas with a majority of white ethnic background, which limits the generalizability of our findings.

## Conclusion

Comorbidities have become increasingly common in patients with HF. Thus, it is important to understand how comorbid conditions influence patients’ decisions about HF self-care. Previous studies have shown that patients with HF and comorbid conditions face challenges with HF self-care because each comorbid condition presents competing demands. Our study expands previous findings. We found that patients with a greater number of non-cardiovascular comorbidities were at risk for poorer HF self-care, but not for those with a greater number of cardiovascular comorbidities. We also found that this significant relationship did not hold when perceived control and depressive symptoms were considered. Our results suggest the potential mediating effect of perceived control and depressive symptoms on the relationship between HF self-care and comorbidities. However, further research is needed to increase our understanding of these relationships. Studies are also needed to examine whether number and types of comorbidities affect changes in self-care to understand the impact of comorbidities on self-care.

## Data Availability

The datasets used and analysed during the current study are available from the corresponding author on reasonable request.

## List of abbreviations

CI: confidence interval
EHFScB-9: European HF self-care behavior scale-9
HF: heart failure
